# Editorial: Breeding for intercropping

**DOI:** 10.3389/fpls.2023.1143653

**Published:** 2023-02-28

**Authors:** Diego Rubiales, Jérôme Enjalbert, Pierre Hohmann, Niels P.R. Anten, Martin Weih

**Affiliations:** ^1^ Institute for Sustainable Agriculture, CSIC, Cordoba, Spain; ^2^ AgroParisTech, UMR GQE-Le Moulon, Institut National de Recherche pour l’agriculture, l’alimentation et l’environnement (INRAE), Gif-sur-Yvette, France; ^3^ BETA Technological Center, University of Vic, Vic, Spain; ^4^ Centre for Crop Systems Analysis, Wageningen University and Research, Wageningen, Netherlands; ^5^ Department of Crop Production Ecology, Swedish University of Agricultural Sciences, Uppsala, Sweden

**Keywords:** plant breeding, intercropping, selection, modelling, quantitative prediction

Intercropping, also known as mixed cropping, consists on simultaneously growing more than one species on a field. It has a great potential for enhancing water- and nutrient-use efficiency and improving plant productivity, yield stability and resilience to biotic and abiotic stress, including those triggered by climate change. Despite their manifold benefits, the practice of intercropping has not risen above its niche status in many regions of the world. The selection of varieties specifically adapted to intercropping remains a major practical challenge to its widespread deployment. This Research Topic hosted at Frontiers in Plant Sciences entitled “Breeding for intercropping” gathers a series of articles covering new insights in the areas of quantitative genetics, ecology, ecophysiology and agronomy integrating theoretical, experimental as well as participatory approaches.

## Why is specific breeding needed for intercrops?

Moutier et al. showed that the performance of genotypes grown in pure stand as monocrops is not necessarily a good indicator for their performance grown in intercrops. In this research performed in France, eight wheat genotypes and five legume testers (three pea and two faba bean varieties) were field-grown as monocrops and in all possible binary intercrops in nine contrasting environments for three years. The mixing abilities of the varieties investigated was evaluated in terms of their ability to maintain or exceed their monocrop yield when grown in intercropping (producer effect); and their ability to benefit the yield of the companion crop (associate effect). Mixing abilities varied greatly between the investigated varieties, both for the wheat and the legume testers, implying that the choice of the legume tester is important for better discriminating the producer or associate effects of the wheat genotype that it is intercropped with. The authors conclude that both the wheat varietal choice and the identity of the legume tester variety are key issues in the breeding for intercropping. They also note that the breeding should consider the mixing ability in terms of both the producer and the associate effects.

In their review, Moore et al. provide an overview of three case studies, identifying relevant considerations for plant breeding efforts. Forage mixtures are the most mature among the cropping systems discussed. However, there is a need to accelerate efforts to breed for mixture systems, e.g., through genomic selection and/or selection of both component species. Breeding for perennial groundcover systems and winter oilseed systems is much less developed. In both cases, there is an opportunity to design a breeding pipeline that incorporates intercropping systems as one of its primary goals. Although nascent, breeding for intercropping systems holds great potential for improving intercropping systems and realizing the potential of this crop diversification strategy for addressing sustainability challenges.

Bourke et al. highlight the need for re-designing breeding programs to accommodate inter-specific interactions, as genotypes bred for monoculture are not the best adapted to intercropping systems. They summarize how to decipher plant interactions in intercropping, studying trait plasticity or plant-microbiome interactions, or exploring its ecophysiological basis using a functional structural plant model (FSPM). They then identify two general breeding strategies, either i) ideotype-driven (i.e., “trait-based” breeding) or ii) quantitative genetics-driven (i.e., “product-based” breeding), and they highlight the interest of the theoretical framework of direct genetic effect (DGE, equivalent to producer effect) and indirect genetic effects (IGE, equivalent to associate effect). They propose a “Powerful Troika”, combining the two strategies, for example coupling FSPM modelling with genomic-assisted selection and analysis of indirect genetic effects.

Current breeding programs do not select for enhanced general mixing ability (GMA) and neglect biological interactions within species mixtures. To address this issue, Haug et al. proposed a model framework for general and specific mixing ability (GMA and SMA). Incomplete factorial designs show the potential to drastically improve genetic gain by providing similar estimates for GMA and SMA variances compared with a two commonly used full factorial designs that employ the same amount of resources. This model was extended to the producer and associate concept to exploit information on fraction yields and allowed to characterise genotypes for their contribution to total mixture yield. Correlations between Producer/Associate effects and plant traits allowed to describe biological interaction functions (BIF) such as commensalism, competition and others. BIF can be used to optimize species ratios at harvest as well as to extend our understanding of competitive and facilitative interactions in a mixed plant community. This study provides an integrative methodological framework to promote breeding for mixed cropping.

Timaeus et al. evaluated inter- and intraspecific diversity intercropping 15 wheat cultivars with one winter pea cultivar under organic conditions. Mixtures increased cereal grain quality, weed suppression, resource-use efficiency, yield gain, and reduced lodging. Under higher nutrient availability, entry-based variation was reduced in both systems, and pea was suppressed. Heterogeneous populations were more stable than line cultivars. Trait analysis revealed a possible link between harvest index and reduced competition in mixture, which can increase yield performance in specific line cultivars. They conclude that while cultivar breeding for mixtures can be successful in monocultures, high environmental variation highlights the necessity of evaluating cultivars in mixtures. In addition, use of intraspecific diversity within interspecific mixed cropping systems can be a valuable addition to further improve mixture performance and its stability under increasing environmental stresses due to climate change.

## Which suits of traits are most important in the breeding for intercropping?

The primary focus of the work by Kiær et al. is the importance of the end use in the evaluation of potential beneficial effects of intercrops. Thus, this perspective paper evaluated breeding targets for genotypes to be grown in intercropping in a supply chain perspective, using three case studies of intercropped legume and cereal species for human consumption to identify crop traits that could be desirable for different actors along the corresponding supply chains. The authors concluded that the widespread adoption and integration of intercrops will only be successful if all supply chain actors are included and collaborate; if the breeding approach takes into account the relative complexity of intercrop supply chains; and if diversification strategies are implemented in every process from field to fork.

Morphological and functional plant traits involved in species interactions were addressed by Peng et al., who evaluated the effects of intercropping on the medicinal plant *Atractylodes lancea* on various morphological traits including growth and volatile oil content. In their field study carried out in a subtropical environment in China, the authors have grown *A. lancea* plants in monocrop and intercropped with with *Zea mays*, *Tagetes erecta*, *Calendula officinalis*, *Glycine max*, or *Polygononum hydropiper* as mixing component. Significantly enhanced growth and accumulation of some volatile oils was found especially when *A. lancea* was mixed with *Z. mays*, *T. erecta* or *C. officinalis*. However, large and significant variation in all measured traits was found also between the two years of this study, and the effects of the mixing treatments on the assessed traits partly varied greatly between the two years; suggesting strong management (here mixing partner) by environment interaction.

Kammoun et al. hypothesized that the grain yield achieved by a cultivar in low nitrogen input durum wheat–grain legume intercrops could be estimated using a few simple variables: (i) the yield of the wheat cultivar at full density in monocrop, (ii) the yield of the legume cultivar at half density in monocrop, and (iii) an indicator of legume cultivar response to interspecific competition that reveals cultivars’ competitive abilities and tolerance to competition. Such a competition index appeared less predictable for the legume than for the durum wheat. Further studies on more diverse genotypes and growing conditions are needed to improve the predictive quality of the model. Moreover, further mechanistic understanding is required to better evaluate the links between the tolerance to interspecific interactions and the plant phenotype characteristics (traits).

Land Equivalent Ratio (LER), is a classical agronomical index used for comparing the performance of species when intercropped, taking as reference their yields in monocrops. Tavoletti and Merletti. proposed to use LER to identify best performing varieties. They explored the yield response to intercropping of durum wheat (*Triticum turgidum* ssp. *durum*) and faba bean (*Vicia faba*), using, respectively, 13 and 3 varieties of the two species. They focussed on a factorial design of 24 mixtures (12 wheat x 2 faba varieties), recording yield in a field trial performed over two years. They observed contrasting performances between the two years, with LER significantly higher than 1 only in the first year. To better discriminate the varietal performances in intecropping, the authors performed principal component and cluster analyses for total yield, LERtotal, i.e. LERw + LERfb, and ln(LERratio), i.e. (LERw/LERfb). This multivariate analysis provides a way to identify the best variety combinations, while the authors propose to use principal component scores as indices of selection within breeding programs aimed to simultaneously improve intercropped species.

Demie et al. reviewed how the performance of cereal/legume intercropping depends on the genotypes used. Over 69 publications analysed, a subset of 35 of them reported land equivalent ratio (LER), with a mean LER of 1.26. Genotype x cropping system (monocrop/intercrop) interactions were tested in 71% of the 69 publications, and reported significant in 75%, of the studies. Interestingly, the different species analysed exhibited different land-use efficiencies in the different design types with finger millet having the highest land-use efficiency for cereals. In most of the studies, the link between traits and intercropping performance were not properly addressed, even if some key traits for intercropping performance, such as earliness, plant height, or growth habit, were also critical in intercropping. The lack of data on traits and genotypes effects on intercropping performance calls for additional experimental efforts, including more genotypes, to improve breeding and blending designs for intercropping systems.

## Can crop growth models and quantitative predictions assist breeding for intercropping?

In a perspective paper, Weih et al. revisited the challenges associated with breeding for intercropping, and gave an outlook on the application of crop growth models to assist breeding for intercropping. Previous approaches using crop models to assist plant breeding were mostly based on the performance and properties of monocrops. For models to be effective in assisting breeding for intercropping, they need to (i) incorporate the relevant plant features and mechanisms driving interspecific plant–plant interactions in the model; (ii) rely on parameters that are closely linked to the traits that breeders would select for; and (iii) be calibrated and validated with field data that are assessed in intercrops. In addition, due to their lower complexity and much reduced parameter requirement, the authors consider minimalist crop growth models to be more likely to incorporate the above elements than comprehensive and parameter-rich crop growth models.

Firmat and Litrico. point out that obtaining reliable community level quantitative predictions for diverse crop systems empirically is limited by the size and complexity of experiments that would be needed. Breeding strategies should instead be compared using theoretically informed qualitative predictions. To this end, they reviewed different approaches arising from the field of evolutionary ecology focusing on: (i) the community heritability approach, (ii) the joint-phenotype approach and (iii) the community trait genetic gradient approach. They suggest research strategies related to each of these approaches.

To explore the interest of genomic selection for intercrop breeding, Bančič et al. proposed an elegant study based on stochastic simulation, where they compared four breeding programs implementing genomic selection and one breeding program based on phenotype only. The different breeding schemes were sized according to a constant budget, using realistic steps as double-haploid production, or intercropping evaluation using testers. Three different genetic correlations (0.4, 0.7, and 0.9) between monocrop and intercrop grain yield were assumed, and only GMA was simulated. Under these three scenarios, all four simulated breeding programs using genomic selection produced significantly more intercrop genetic gain than the phenotypic selection program (∼1.3–2.5 times), but at the cost of genetic variance. Under low genetic correlations, the Grid-GS program, which employed an incomplete factorial instead of using testers, was the most efficient. Authors suggest a genomic selection strategy which combines monocrop and intercrop trait information, using a selection index that includes economic weights, in order to increase selection accuracy.

Annicchiarico et al. studied efficiency of several phenotypic or genomic selection strategies in pea breeding for intercropping with cereals. The efficiency of an indirect selection index including onset of flowering, plant height, and grain yield in monocrops was comparable to that of pea yield selection in intercrops. Genomic selection for pea yield in monocrop displayed an efficiency close to that of phenotypic selection for pea yield in intercrop, and nearly two-fold greater efficiency when also taking into account its shorter selection cycle and smaller evaluation cost.

Instead of breeding to improve monoculture yield of single crops in isolation, Wolfe et al. propose optimizing multiple interacting species and genotypes by enabling joint-selection to improve the performance of the cropping system across time and space. Genomic and phenomic prediction poses an exciting opportunity to develop a multi-tiered selection scheme. There are multiple levels or “tiers” of selection, which when considered jointly enact agroecosystem improvement. The objective at Tier 1 is intraspecific population improvement, which is addressed simultaneously across each species to affect co-adaptation of the germplasm pools. At Tier 2, selection is focused on predictions of performance of the combination over space and time.

The practice of wheat variety mixtures is spreading. However, there are few blending rules to design variety mixtures, and not any based on plant architecture. As the high dimensionality of trait combinations in intercopping is hardly compatible with field experiments, Blanc et al. proposed to use the FSPM WALTer to simulate wheat cultivar mixtures and try to better understand how key traits driving the aerial architecture can influence mixture performance. However, most FSPM are slow to run and do not allow to explore the combinatorics of their numerous parameters. Hence the authors combined two original methods: i) they used a metamodel of WALTer, i.e. an approximation of the FSPM outputs, to speed up computation, and ii) they then performed a sensitivity analyses based on both mean and differences in architectural trait values of the mixed components (binary and balanced mixtures). These analyses highlighted the impact of the leaf dimensions and the tillering capability on the performance of the simulated mixtures. Identifying the best performing mixtures revealed original combinations of ideotypes with contrasting tillering abilities and leaf dimensions, asking for experimental confirmations.

## Author contributions

All authors listed have made a substantial, direct, and intellectual contribution to the work and approved it for publication.

